# Modified OPTIModel with oligometastatic disease for the prediction of overall survival of patients with renal cell cancer and symptomatic long bone metastases

**DOI:** 10.1016/j.jbo.2025.100709

**Published:** 2025-09-02

**Authors:** E.W. Dootjes, J.J. Willeumier, C.W.P.G. van der Wal, R.J.P. van der Wal, P. van der Zwaal, A. Leithner, A.A.M. van der Veldt, M. Fiocco, D.L.M. van Broekhoven, Y.M. van der Linden

**Affiliations:** a Department of Orthopaedics and Sport Medicine, Erasmus Medical Centre, Rotterdam, the Netherlands; b Department of Orthopaedic Surgery, Leiden University Medical Centre, Leiden, the Netherlands; c Department of Orthopaedic Surgery, Haaglanden Medical Centre, The Hague, the Netherlands; d Department of Orthopaedics and Trauma, Medical University of Graz, Graz, Austria; e Department of Medical Oncology, Erasmus Medical Centre, Rotterdam, the Netherlands; f Department of Radiology and Nuclear Medicine, Erasmus Medical Centre, Rotterdam, the Netherlands; g Medical Statistics Department of Biomedical Data Sciences, Leiden University Medical Centre, Leiden, the Netherlands; h Mathematical Institute, Leiden University, Leiden, the Netherlands; i Department of Solid Tumors, Princess Máxima Center for Pediatric Oncology, Utrecht, the Netherlands; j Department of Radiotherapy, Leiden University Medical Centre, Leiden, the Netherlands; k Netherlands Comprehensive Cancer Organisation (IKNL), Utrecht, the Netherlands

**Keywords:** Renal cell carcinoma, Bone metastases, Symptoms, Survival, Prediction

## Abstract

**Aims:**

For patients with long bone metastasis (LBM), we have previously developed OPTIModel. In this study, we investigated whether the OPTIModel could be improved for patients with metastatic renal cell cancer (mRCC) by including oligometastatic bone metastases (OBM) as a risk factor.

**Methods:**

Patients with mRCC and symptomatic LBMs were included in a retrospective and prospective multicenter cohort. Bone metastases (BMs) were categorized as: solitary (SBM), limited BMs (2–4 BMs) or diffuse BMs (DBM; >4 BMs). OBM were defined as ≤ 4 BMs. Overall survival was estimated using Kaplan Meier method. Effect of risk factors on overall survival were assessed using multivariate Cox regression model. Based on these results, the OPTIModel was modified. To assess the discriminatory ability, Harrell’s C-statistic was used.

**Results:**

178 patients were included. Overall, median overall survival was 12.1 months (95 % confidence interval (CI): 8.8–15.3). Median survival for SBM (n = 53, 29.8 %), limited BMs (n = 60, 33.7 %) and DBMs (n = 65, 36.5 %) was 19.6 months (95 %CI: 6.8–32.4), 14.8 months (95 %CI: 7.6–21.9) and 6.1 months (95 %CI: 2.7–9.5), respectively. Median survival was 16.3 months (95 %CI: 10.6–22.0) in patients with OBM (n = 113, 63.5 %), with a hazard ratio of 2.11 (95 %CI: 1.44–3.09) compared to patients with DBM. Including OBM in the OPTIModel for mRCC improved C-statistic from 0.585 (standard error (SE) = 0.027) to 0.618 (SE = 0.024).

**Conclusion:**

Both SBM and limited BMs were associated with a longer overall survival in patients with mRCC and symptomatic LBMs. The modified OPTIModel for mRCC with inclusion of oligometastatic disease could guide decisions about local treatment of symptomatic LBMs.

## Highlights

• Renal cell carcinoma patients with oligometastastic bone lesions have better survival than those with diffuse bone metastases.
• A survival prediction tool informs clinicians on expected remaining survival.
• The OPTIModel helps clinicians discuss treatment options with patients in light of their prognosis.

## 1 Introduction

Renal cell cancer (RCC) is commonly reported in highly developed countries, with approximately 138.600 new cases and 54.100 deaths in 2020 in Europe [1]. At time of the initial diagnosis, nearly one in three patients has metastatic disease [2]. The most common sites for metastases are lungs (45 %), bones (30 %), and lymph nodes (22 %) [3]. First-line combination therapies of immune checkpoint inhibitors and tyrosine kinase inhibitors (TKIs) have significantly improved the survival of patients with metastatic renal cell carcinoma (mRCC), but patients with bone metastases (BMs) still have a poorer overall survival as compared to patients with any metastasis (35 versus 53 months) [4].

In patients with mRCC, long bone metastases (LBMs) have a high fracture risk and significant impact on quality of life [5]. Local treatment options of LBMs include surgery (en bloc or intralesional resection) and radiotherapy (both conventional low dose or high precision high dose). However, determining the local treatment for patients with mRCC can be challenging. Eligibility for both surgery or radiotherapy mainly depends on the presence of an actual or impending fracture and patients’ expected survival [6]. Historically, survival estimation for patients with symptomatic LBMs was mainly based on expert opinion. More recently, prognostic models to estimate survival have been developed to guide decision making in patients with BMs [7,8].

**Fig. 1:**
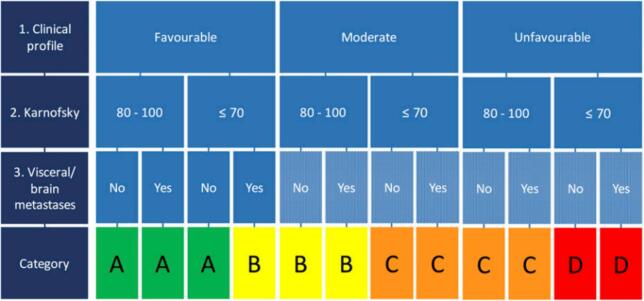
OPTIModel as developed by Willeumier JJ et al.^10^.

For patients with LBMs, we have previously developed the OPTIModel which is based on the primary tumour histology, the Karnofsky Performance Score (KPS) [9] and the presence of visceral and/or brain metastases (VBM)(Fig. 1) [10]. The OPTIModel is an easy-to-use survival estimation model and is used in clinical practice in the Netherlands in accordance with the Dutch guidelines for patients with BMs. To further improve the OPTIModel for patients with different solid tumours, disease specific prognostic factors were incorporated in the model for patients with lung and breast cancer [11,12].

In patients with RCC, solitary bone metastasis (SBM) is considered a favourable prognostic factor and was already incorporated in the current OPTIModel [10]. Nowadays, the term oligometastatic disease is increasingly used and oligometastatic bone metastases (OBM) are defined as a maximum of four BMs [13]. Patients with oligometastatic disease usually have a longer survival than patients with diffuse bone metastases (DBM) [[14], [15], [16]]. Therefore, more extensive treatments, e.g. ablative stereotactic body radiotherapy or even en bloc resections, could not only be considered for SBM, but also for limited BMs (2–4 BMs) [17,18].

In this study, we investigated whether the prognostic value of the OPTIModel could be improved for patients with mRCC and symptomatic LBMs by including oligometastatic disease as a risk factor. Therefore, the prognostic factor for metastatic burden in bone was modified for oligometastatic disease and incorporated in the OPTIModel.

## 2 Patients and Methods

### 2.1 Study design

For the development of OPTIModel a multicentre retrospective database was previously created to collect survival data of patients with symptomatic LBMs [10]. Between 2000 and 2014, all consecutive patients were included when they were treated for LBMs at the departments of orthopaedic surgery or radiotherapy in six hospitals (Leiden University Medical Centre, Erasmus Medical Centre, University Medical Centre Utrecht, University Medical Centre Groningen, Haaglanden Medical Centre and Reinier de Graaf Hospital) in the Netherlands. The OPTIModel was validated with an external cohort (Medical University of Graz) [10]. In addition, a multicentre prospective database (ProMISe) was created. In this prospective cohort, patients who were treated for symptomatic LBMs between 2012 and 2020 were included in four Dutch hospitals (Leiden University Medical Centre, Erasmus Medical Centre, Haaglanden Medical Centre and University Medical Centre Groningen).

For all cohorts of the OPTIModel, the external validation and the ProMISe, the collected following clinical variables included primary tumour histology, date of diagnosis of the primary tumour and the presence of symptomatic BMs, number of BMs, presence of VBM, KPS, systemic treatment, use of TKIs and local treatment of symptomatic LBMs**.**

### 2.2 Data collection

For this study, all patients with mRCC were selected from the cohorts of OPTIModel, external validation and ProMISe. First, bone metastases were categorized into three categories: SBM (solitary bone metastasis), limited number of BMs (2–4 bone metastases) or DBM (>4 bone metastases). To align with current literature, we then combined SBM and limited BMs into a group of OBM [13]. To determine the number of BMs, all available radiology reports were reviewed, including reports of magnetic resonance imaging (MRI), computed tomography (CT), positron emission tomography (PET)/CT and bone scintigraphy. In the OPTIModel cohort, patients were only included if radiology reports were available within three months before or after initial local treatment for symptomatic LBMs. Only reports available within three months before or after initial local treatment for symptomatic LBMs were used. Reports before or after this period were only used if both pre- and posttreatment reports described the same number of BMs, pretreatment reports described > 4 BMs or post-treatment reports described SBM. In the prospective ProMISe cohort, the total number of BMs was registered at the time of initial treatment of a symptomatic LBM. To investigate the relationship between the period from diagnosis of primary RCC to treatment for symptomatic BMs, the symptomatic bone metastatic interval (SBMI) was calculated.

Performance status was scored according to the KPS. In case of the Eastern Cooperative Oncology Group (ECOG) and WHO scores were reported, these scores were converted into KPS. According to the KPS, patients were categorized into two groups: ≥ 80 (able to continue normal activities) and ≤ 70 (unable to continue normal activities).

The presence of visceral metastases was based on radiology reports. The presence of brain metastases was based on whole brain CT or MRI, if available. If brain imaging was not available, it was assumed that brain metastases were absent.

### 2.3 Modelling

**Fig. 2:**
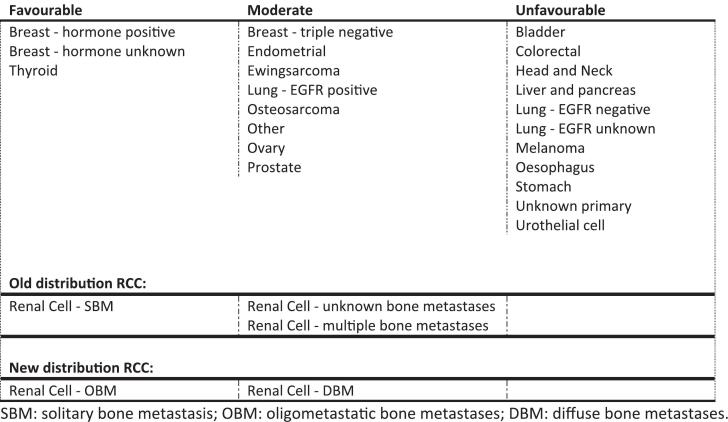
Old and new distribution of primary tumours into the different clinical profiles in the OPTIModel. SBM: solitary bone metastasis; OBM: oligometastatic bone metastases; DBM: diffuse bone metastases.

In the OPTIModel (Fig. 1), primary tumours are categorized according to three clinical profiles; favourable, moderate and unfavourable (Fig. 2). Specifically for RCC, SBM is defined as a favourable risk factor, whereas having more BMs is defined as a moderate risk factor [10]. This subdivision was created based on literature available at the time the OPTIModel was developed [12]. To investigate whether the OPTIModel could be further improved, the factor SBM (one BM) was replaced by the factor OBM (≤4 BMs) and the definition of DBM (>4 BMs) was adapted accordingly.

### 2.4 Statistics

To test differences between the groups, Kruskal-Wallis non-parametric test for continuous variables and Chi-Square for categorical data were used. Time of overall survival was calculated from date of first local treatment for symptomatic LBM to date of death or last follow-up. The Kaplan-Meier method was used to estimate survival and the Log Rank test was used to assess the difference in overall survival between SBM, OBM and DBM. Median follow up was estimated with reversed Kaplan-Meier’s methodology [19]. Univariate and multivariable Cox proportional hazards regression model were estimated to study the association between overall survival and the year of diagnosis of the primary tumour, KPS, presence of VBM, the number of BMs, fracture status, lesion location, age at local treatment, treatment with TKIs and SBMI. The discriminatory ability of the OPTIModel and modified OPTIModel was assessed using Harrell’s C-statistic [20]. Significance level was set at 0.05, statistical analyses were performed by using SPSS 24.0 (SPSS Inc., Armonk, NY) and in the R software environment by using the survival library.

## 3 Results

### 3.1 Patient characteristics

A total of 214 patients with mRCC and symptomatic LBM were identified. In 36 patients, the reports did not fulfil the requirements. After exclusion of these patients, 178 patients could be included for the analyses (Table 1). Most patients were male (69.1 %) and the median age at time of first local treatment for LBMs was 68.0 years (interquartile range (IQR): 59.9–74.5). The median SBMI was 1.0 years (IQR: 0.1–4.6). Ninety-five (53.4 %) patients had a KPS score of ≥ 80. In 105 (59.0 %) patients, VBM was present and 49 (27.5 %) patients were treated with TKIs. For LBMs, the femur was the primary site of first local treatment in 98 (55.1 %) patients. In addition, 78 (43.8 %) patients had actual pathological fractures and 61 (34.3 %) patients had impending fractures. Of these 139 patients, 107 patients underwent surgical intervention as their first treatment of whom 64 patients received postoperative radiotherapy. Of the 61 patients with impending fractures, 46 (25.8 %) patients were treated with radiotherapy alone to palliate pain and prevent fracturing. According to the number of BMs, 53 (29.8 %) patients had SBM, 60 (33.7 %) patients had limited BMs and 65 (36.5 %) patients had DBM. In these groups, gender, age at local treatment of the BMs, SBMI, KPS, local treatment and fracture status were similar (Table 1). The median SBMI for SBM, limited BM and DBM was 1.8 years (IQR: 0.1–8.5), 0.9 years (IQR: 0.2–3.4) and 1.2 years (IQR: 0.2–4.6), respectively.

**Table 1:** Patient and treatment characteristics, N (%).

Characteristics	Included	SBM	Limited BM	DBM
**Patients**	178 (100)	53 (29.8)	60 (33.7)	65 (36.5)
**Gender***^§^*, male	123 (69.1)	38 (71.7)	40 (66.7)	45 (69.2)
**Age at first treat BM***^§^*, median (IQR), years	68.0 (59.9–74.5)	68.4 (58.9–75.3)	69.2 (60.6–75.6)	66.6 (59.9–73.5)
**SBMI***^§^*, median (IQR), years	1.0 (0.1–4.6)	1.6 (0.1–7.8)	0.9 (0.1–3.4)	0.8 (0.1–4.6)
**KPS***^§^*				
80–100	95 (53.4)	30 (56.6)	35 (58.3)	30 (46.2)
0–70	58 (32.6)	17 (32.1)	18 (30.0)	23 (35.4)
Missing	25 (14.0)	6 (11.3)	7 (11.7)	12 (18.5)
**VBM***^§^*				
Present	105 (59.0)	30 (56.6)	38 (63.3)	37 (56.9)
Not present	73 (41.0)	23 (43.4)	22 (36.7)	28 (43.1)
**Treatment primary tumour***^§^*				
Surgery only	100 (56.2)	37 (69.8)	35 (58.3)	28 (43.1)
Systemic only	40 (22.5)	7 (13.2)	15 (25.0)	18 (27.7)
Combination	13 (7.3)	4 (7.5)	3 (5.0)	6 (9.2)
No treatment	23 (12.9)	5 (9.4)	6 (10.0)	12 (18.5)
Missing	2 (1.1)	0	1 (1.7)	1 (1.5)
**TKI***^§^*				
Yes	49 (27.5)	12 (23.1)	18 (30.0)	19 (27.7)
No	128 (71.9)	40 (76.9)	42 (70.0)	46 (72.3)
**Localisation***^§^*				
Femur	98 (55.1)	32 (60.4)	31 (51.7)	35 (53.8)
Humerus	57 (32.0)	13 (24.5)	17 (28.3)	27 (41.5)
Tibia	11 (6.2)	3 (5.7)	7 (11.7)	1 (1.5)
Other	12 (6.7)	5 (9.4)	5 (8.3)	2 (3.1)
**Fracture status***^§^*				
Actual	78 (43.8)	27 (50.9)	25 (41.7)	26 (40.0)
Impending	61 (34.3)	13 (24.5)	21 (35.0)	27 (41.5)
No fracture	39 (21.9)	13 (24.5)	14 (23.3)	12 (18.5)
**Local treatment metastasis***^§^*				
Surgery only	43 (24.2)	19 (35.8)	14 (23.3)	10 (15.4)
Surgery + RT	64 (36.0)	22 (41.5)	20 (33.3)	22 (33.8)
RT + surgery	25 (14.0)	3 (5.7)	13 (21.7)	9 (13.8)
RT only	46 (25.8)	9 (17.0)	13 (21.7)	24 (36.9)
**Enbloc surgery performed**				
Yes	24 (13.5)	17 (32.1)	6 (10.0)	1 (1.5)
No	154 (86.5)	36 (67.9)	54 (90.0)	64 (98.5)

SBM: ‘solitary bone metastasis’; BM: ‘bone metastases’; DBM:’diffuse bone metastases’; SBMI: ‘symptomatic bone metastases interval’; IQR: ‘Interquartile range’; KPS: ‘Karnofsky performance score’; VBM: ‘visceral − and / or brain metastases’; TKI: ‘tyrosine kinase inhibitor’; RT:’radiotherapy’. *^§^No significant difference between groups was observed.*

### 3.2 Bone metastases and survival

**Fig. 3:**
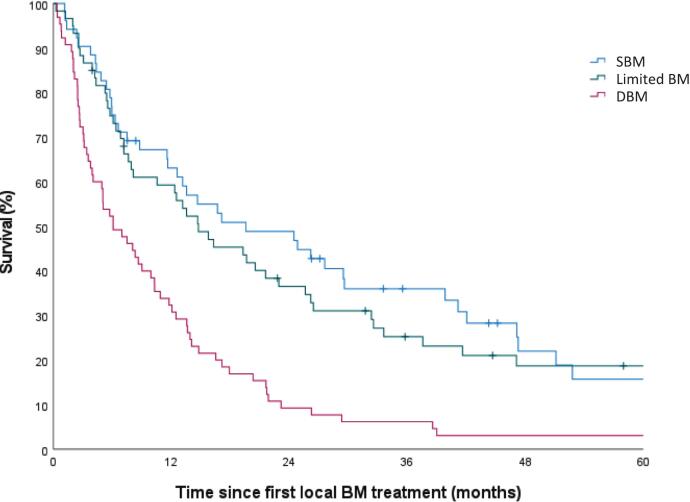
Estimated overall survival of 178 renal cell carcinoma patients with symptomatic bone metastases by metastatic status (‘SBM’ n = 53; ‘limited BM’ n = 60; ‘DBM’ n = 65 (p < 0.001)). SBM: solitary bone metastasis; limited BM: limited bone metastases; DBM: diffuse bone metastases.

**Fig. 4:**
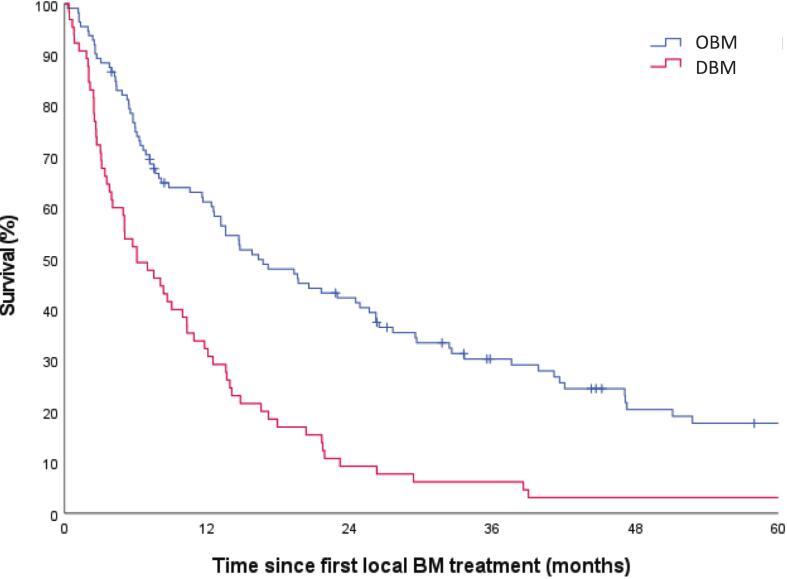
Estimated overall survival of 178 renal cell carcinoma patients with symptomatic bone metastases by metastatic status (‘OBM’ n = 113; ‘DBM’ n = 65 (p < 0.001)). OBM: oligometastatic bone metastases; DBM: diffuse bone metastases.

Median overall survival was 12.1 months (95 % confidence interval (CI): 8.8–15.3) with a median follow-up of 7.1 years (range 0.3–14.7). Median overall survival time was 19.6 months (95 %CI: 6.8–32.4), 14.8 months (95 %CI: 7.6–21.9) and 6.1 months (95 %CI: 2.7–9.5) for SBM, limited BMs and DBM, respectively (Fig. 3). Median overall survival for the combined OBM group, including patients with SBM and limited BMs, was 16.3 months (95 %CI: 10.6–22.0) (Fig. 4). Hazard ratio (HR) for DBM was equal to 2.11 (95 %CI: 1.44–3.09); reference category combined OBM group. Two years survival was 42.3 % (95 %CI: 32.9–51.7) in patients with OBM and 9.2 % (95 %CI: 2.1–16.3) in patients with DBM. Multivariable analyses showed significant differences in overall survival for KPS (HR = 1.62, 95 %CI: 1.12–2.33), VBM (HR = 1.71, 95 %CI: 1.17–2.50) and en bloc surgery (HR = 0.49, 95 %CI: 0.27–0.88) (Table 2).

**Table 2:** Univariate and multivariate Cox proportional hazards regression model for overall survival.

Item	UV	MV				
**Metastatic load**	HR	95 % CI	p-value	HR	95 % CI	p-value
SBM + limited BMs	ref			ref		
DBM	**2.36**	**1.69-3.28**	**< 0.001**	**2.11**	**1.44**–**3.09**	**<0.001**
**Age at treatment BMs**						
<65	ref			ref		
≥65	1.31	0.94–1.82	0.11	1.19	0.82–1.72	0.35
**SBMI**						
<60 months	ref			ref		
≥60 months	1.02	0.70–1.49	0.92	1.2	0.76–1.89	0.44
**KPS**						
≥80	ref			ref		
≤70	**1.87**	**1.31**–**2.67**	**< 0.001**	**1.62**	**1.12– 2.33**	**0.01**
**VBM**						
No	ref			ref		
Yes	**1.47**	**1.05**–**2.05**	**0.02**	**1.71**	**1.17**–**2.50**	**0.01**
**TKI**						
Yes	ref			ref		
No	1.16	0.81–1.66	0.43	1.2	0.78–1.85	0.41
**Localisation**						
Upper extremity	ref			ref		
Lower extremity	0.96	0.69–1.33	0.8	0.88	0.60–1.28	0.51
**Pathological fracture**						
No	ref			ref		
Yes	1.06	0.77–1.47	0.71	1.01	0.69–1.47	0.98
**Enbloc surgery**						
No	ref			ref		
Yes	**0.39**	**0.23 –0.67**	**< 0.001**	**0.49**	**0.27**–**0.88**	**0.02**

UV: ‘univariate analyses’; MV: ‘multivariate analyses’; HR: ‘hazard ratio’; CI: ‘confidence interval’; SBM: ‘solitary bone metastasis’; BMs: ‘bone metastases’; DBM:’diffuse bone metastases’; SMBI:’symptomatic bone metastasis interval’; KPS: ‘Karnofsky performance score’; VBM: ‘visceral and / or brain metastases’; TKI: ‘tyrosine kinase inhibitor’.

### 3.3 Modified OPTIModel

For patients with mRCC and BMs, the OPTIModel was adapted according to the number of BMs. Having ≤ 4 BMs (OBM) was categorised as a favourable risk factor, whereas the presence of > 4 BMs (DBM) was a moderate risk factor (Fig. 2). For the modified model, results are shown in Table 3. After incorporation of oligometastatic BMs in the OPTIModel, the C-statistic increased from 0.585 (standard error (SE) = 0.027) to 0.618 (SE = 0.024), suggesting a small and non-significant improvement in the discriminatory ability of the model.

**Table 3:** Before and after modification of ^§^categories A to D based on OPTIModel (Fig. 1).

PredictiveCategory	n (%)	Median Overall Survival, months (95 % CI)	HR	95 % CI	C-statistic
Original OPTIModel [10]					**0.58**
A	36 (23.5)	26.2 (15.4–37.0)	ref	ref	
B	76 (49.7)	10.5 (5.9–15.2)	1.76	1.10–2.82	
C	41 (26.8)	7.6 (2.5–12.7)	2.23	1.33–3.75	
D	NA	NA	NA	NA	

Modified adjustment					**0.62**
A	78 (51.0)	21.6 (12.1–31.0)	ref	ref	
B	52 (34.0)	7.2 (5.3–9.0)	2.11	1.43–3.12	
C	23 (15.0)	5.7 (1.0–10.4)	2.86	1.74–4.70	
D	NA	NA	NA	NA	

CI: ‘confidence interval’; NA: ‘not applicable’ (no patients in this category).

## 4 Discussion

In this study, we investigated impact of the number of BMs on overall survival in patients with mRCC and symptomatic LBMs. Patients with symptomatic LBMs and oligometastatic bone metastases (≤4 BMs) had a significantly longer overall survival as compared to patients with diffuse bone metastases (>4 BMs; 16.3 *versus* 6.1 months). Therefore, oligometastatic disease was added as a factor of mRCC to the OPTIModel, which is an easy-to-use tool to guide the local treatment strategy of symptomatic LBMs. This modified OPTIModel with inclusion of oligometastatic disease is more in line with current clinical practice of mRCC.

There are large differences in disease trajectories for patients with metastatic cancer [21]. Patients with a short SBMI after primary tumour diagnosis tend to have more advanced disease and have a shorter overall survival after the diagnosis of the metastatic disease as compared to patients with a long SBMI [22]. However, in the current study, SBMI was not different between patients with SBM, limited BM and DBM. After adjusting for SBMI, the number of BMs was significantly associated with overall survival, suggesting that the observed difference in overall survival might be due to possible differences in disease burden and tumour biology.

In the current study, 30 % of the patients survived two years or longer. These patients could possibly benefit from more extensive treatment strategies for symptomatic LBMs. More ablative local treatment in patients with SBM and especially limited BM is still debatable. Durable local control could be achieved with en bloc resection often followed by endoprosthetic reconstruction (EPR), and/or high dose radiotherapy (e.g. stereotactic body radiotherapy (SBRT)). Numerous studies have reported the association between metastatic burden of BMs and survival in patients with mRCC [[23], [24], [25], [26], [27], [28], [29], [30]]. Most of these studies focused on survival of patients with mRCC and SBM who were treated with en bloc resection or intralesional resection [24,25,28,29]. In addition, several studies reported an improved survival in patients who were primarily treated with en bloc resection followed by EPR as compared to other types of skeletal stabilization [25,27]. However, these findings may reflect patient selection, instead of the outcome of the local intervention. Since most of these patients had a SBM, it is still unknown whether patients with limited BMs benefit from en bloc resection. Our multivariate analyses suggest a positive effect of en bloc surgery on survival in patients with SBM and limited BMs. However, the results should be interpreted with caution since en bloc surgery was performed in a limited number of patients.

For patients with mRCC who are candidates for extensive surgery, SRBT can still be considered to treat symptomatic LBMs. However, despite excellent 1-year local control and low toxicity, the benefit of SBRT on survival is questionable [31]. Tree et al. reported that 20–30 % of patients met mRCC are progression free at five years follow up after SBRT treatment for oligometastases [13]. To optimize the treatment of LBMs, prognostic models are needed to guide local treatment for patients with mRCC. These models, such as the OPTIModel [10], could guide treating physicians and patients to determine whether local treatment strategies with ablative radiotherapy or en bloc surgery could be beneficial. While the original OPTIModel already differentiated between solitary and multiple BMs from mRCC, this study shows that patients with 2–4 BMs also have a more favourable outcome (Table 3). These findings suggest that patients with OBM (1–4 lesions), KPS ≥ 80, and absence of VBM could be eligible for radical local treatment to improve and prolong mobility, thereby improving their quality of life.

Bone metastases from RCC are usually lytic and prone to skeletal related events (SREs), which are defined as pathological fractures, bone surgery for pending pathological fractures, bone pain requiring radiotherapy, spinal cord and nerve root compression, and hypercalcaemia. These SREs are reported in approximately 80 % of patients with RCC and bone metastases [32]. In addition to surgery and radiotherapy, bone modifying agents, such as bisphosphonates and denosumab, can be administered to reduce the risk of SREs. In patients with mRCC and BMs, bisphosphonates can reduce the SRE rate with 55 % when combined with radiotherapy [33]. Therefore, bone modifying agents can be considered as a component of multimodal therapy strategy in patients with mRCC and BMs. However, since the Dutch guidelines did not recommend the use of bisphosphonates for patients with mRCC, we could not not include bisphosphonates in the analyses.

A limitation of the study is the retrospective design and the use of different imaging modalities. In mRCC, PET/CT using ^18^F-FDG has a higher sensitivity for the detection of BMs as compared to CT or bone scintigraphy [34], resulting in a potential underestimation of metastatic burden in some patients [35]. In addition, the number of BMs could be underestimated due to different field of views of the different imaging modalities.

In addition, the long inclusion period and the lack of systemic treatments in this period may have limited further optimalization of the modified OPTIModel. Between 2000 and 2014, the first TKIs targeting VEGFR signalling were introduced in the Netherlands. After 2014, immune checkpoint inhibitors and several combinations with immune checkpoint inhibitors were approved in the Netherlands for the treatment of mRCC. With the increasing number of available systemic treatments, the use of the risk model of the International Metastatic RCC Database Consortium (IMDC) has increased to select patients for the different systemic treatments. However, the IMDC risk factors (e.g. hypercalcaemia and neutrophilia) were not collected in the current study. Nevertheless, the data of the used homogenous cohorts with long follow-up are still useful since the clinical outcomes in these cohorts were not significantly affected by the different systemic strategies that are currently available in clinical practice. Therefore, the modified OPTIModel may still be valuable to guide local treatment of symptomatic LBMs in the current era of mRCC treatment.

In conclusion, both solitary bone metastasis and limited (2–4) bone metastases were associated with a longer overall survival in patients with mRCC and symptomatic LBMs. The modified OPTIModel with inclusion of oligometastatic disease may assist treating physicians and patients in making decisions about local treatments of symptomatic LBMs, thereby improving the quality of life of patients with mRCC and symptomatic LBMs.

## CRediT authorship contribution statement

**E.W. Dootjes:** Writing – original draft, Visualization, Formal analysis, Data curation. **J.J. Willeumier:** Writing – review & editing, Resources. **C.W.P.G. van der Wal:** Writing – review & editing, Resources. **R.J.P. van der Wal:** Writing – review & editing, Resources. **P. van der Zwaal:** Writing – review & editing, Resources. **A. Leithner:** Writing – review & editing, Resources. **A.A.M. van der Veldt:** Writing – review & editing. **M. Fiocco:** Writing – review & editing, Validation, Methodology, Formal analysis. **D.L.M. van Broekhoven:** Writing – review & editing. **Y.M. van der Linden:** Writing – original draft, Validation, Resources, Project administration, Methodology, Funding acquisition, Conceptualization.

## Funding

The OPTIMAL study was supported by a grant from the Dutch Cancer Society / Alpe d’HuZes.

## Declaration of competing interest

The authors declare that they have no known competing financial interests or personal relationships that could have appeared to influence the work reported in this paper.
